# The reaction of sponsor stock prices to clinical trial outcomes: An event study analysis

**DOI:** 10.1371/journal.pone.0272851

**Published:** 2022-09-02

**Authors:** Manish Singh, Roland Rocafort, Cathy Cai, Kien Wei Siah, Andrew W. Lo

**Affiliations:** 1 MIT Laboratory for Financial Engineering, Cambridge, Massachusetts, United States of America; 2 Department of Electrical Engineering and Computer Science, Massachusetts Institute of Technology, Cambridge, Massachusetts, United States of America; 3 MIT Sloan School of Management, Cambridge, Massachusetts, United States of America; 4 Computer Science and Artificial Intelligence Laboratory, Massachusetts Institute of Technology, Cambridge, Massachusetts, United States of America; Children’s Hospital Srebrnjak: Djecja Bolnica Srebrnjak, CROATIA

## Abstract

We perform an event study analysis that quantifies the market reaction to clinical trial result announcements for 13,807 trials from 2000 to 2020, one of the largest event studies of clinical trials to date. We first determine the specific dates in the clinical trial process on which the greatest impact on the stock prices of their sponsor companies occur. We then analyze the relationship between the abnormal returns observed on these dates due to the clinical trial outcome and the properties of the trial, such as its phase, target accrual, design category, and disease and sponsor company type (biotechnology or pharmaceutical). We find that the classification of a company as “early biotechnology” or “big pharmaceutical” had the most impact on abnormal returns, followed by properties such as disease, outcome, the phase of the clinical trial, and target accrual. We also find that these properties and classifications by themselves were insufficient to explain the variation in excess returns observed due to clinical trial outcomes.

## Introduction

Many factors are known to influence the stock prices of companies. They include earnings announcements [1], dividends [2], corporate actions [3], the release of new products [4], etc. For biopharmaceutical companies, apart from these general factors, clinical trial outcomes are critical since they play a major role in determining the future revenue, growth, and valuation of the company sponsoring the trial. For investors in this sector, clinical trial results are the predominant factor determining their return on investment. Therefore, quantifying the impact of clinical trial results on the stock prices of the companies is of great financial significance.

Apart from the goals of investors, however, the study of the movement of stock prices before the release of public information is also relevant for regulators, as an unexpected rise or fall of stock prices just *before* the release of news may be indirect evidence of insider trading [5, 6]. In addition, the study of excess returns after an event can be used to gauge the efficiency of markets. The Efficient Markets Hypothesis states that share prices fully reflect all available information about a company [7]; hence, upon the public release of new information, the price change should reflect this information. Therefore, the excess returns after an event can be used to gauge the degree of market efficiency and the speed with which the market incorporates new information.

On May 18, 2020, Moderna, Inc., announced its positive interim phase 1 trial data for its mRNA vaccine SARS-CoV-2, which led to its stock rising by 35% in a single day. Similarly, when Pfizer announced the interim results of its phase 3 SARS-CoV-2 vaccine, its stock rose by 9.2% on the announcement day. But the impact on stock prices from the phase 3 trial results by Pfizer was considerably smaller than the impact of the release of phase 1 trial data by Moderna. This observation raises multiple questions, including: what impact do clinical trial outcomes have on the stocks of their sponsoring companies? What properties of clinical trials influence these stock prices? And what kind of companies are more affected by the success or failure of a clinical trial? The answers to these questions will assist investors in their management of risk, and allow them to anticipate the impact of clinical trial results on their portfolio.

In this study, we answer these questions by performing an event study analysis for 13,807 clinical trial outcomes using data from 2000 to 2020 for 379 publicly traded U.S. companies. To the best of our knowledge, this is the largest event study analysis of clinical trial outcomes. We analyze the abnormal returns (defined as excess returns relative to a linear factor model described in Section *Methodology*.) for the multiple days around event (the day before the event, the day of event, the day after the event). We restrict our analysis to a short time window around the event to reduce the impact of confounding events from affecting our analysis.

To perform the study, we first identify the dates most relevant to clinical trial announcements on which the largest impact on the stock prices of their sponsor companies occur. Next, we observe the variation in abnormal returns across trials with different outcomes and different trials with same outcome, and investigate the factors responsible for this variation. We then develop a model relating the observed abnormal returns to the factors of a clinical trial, including trial phase, outcome type, target accrual, disease therapeutic ID, trial design, and sponsor company classification. We call this analysis a return attribution model, since it attributes the abnormal returns to various clinical trial factors. We perform this attribution for abnormal returns over the days immediately prior to and after the event. Our model can be used to measure the impact of the results of clinical trials on the returns of their sponsor company by using the multiple factors associated with a clinical trial.

Through this analysis, we obtain several insights. For individual clinical trials, we find that the sponsor company classification had the greatest impact on abnormal returns, with early biotechnology companies having greater movement in stock prices than large pharmaceutical companies when trial results were declared. We also find that factors like phase 2/3 and 3 trials, the disease categories of genitourinary and ophthalmology, and placebo-controlled trials had the largest abnormal returns in their respective categories, while phase 1 and 1/2 trials, the disease categories of autoimmune/inflammation and endocrinology, and multiple ascending dose studies have the minimum abnormal returns in their respective categories. Furthermore, we observe a positive correlation between the total target accrual of the trial and the stock’s abnormal returns. Finally, our abnormal return attribution model finds that clinical trial properties and sponsor company classification were not sufficient to explain differences in the excess returns across different trial outcomes.

Our main contributions can be summarized as follows:

- With 13,807 clinical trial outcomes from 2000 to 2020, the scope of our event study analysis is orders of magnitude larger than earlier studies, yielding more accurate inferences regarding the impact of clinical trial outcomes on sponsor company stock prices.
- We measure the relationship between the properties of clinical trials (phase type, outcome, disease category, target accrual, trial design) and the classification of their sponsor companies as biotech or pharma on the abnormal returns of sponsor company stock, which has not been extensively explored in prior event studies of clinical trials.
- A robust statistical model was developed that estimates the average abnormal returns due to a clinical trial’s outcome given the properties of the clinical trial and its sponsor company.

**Fig 1:**
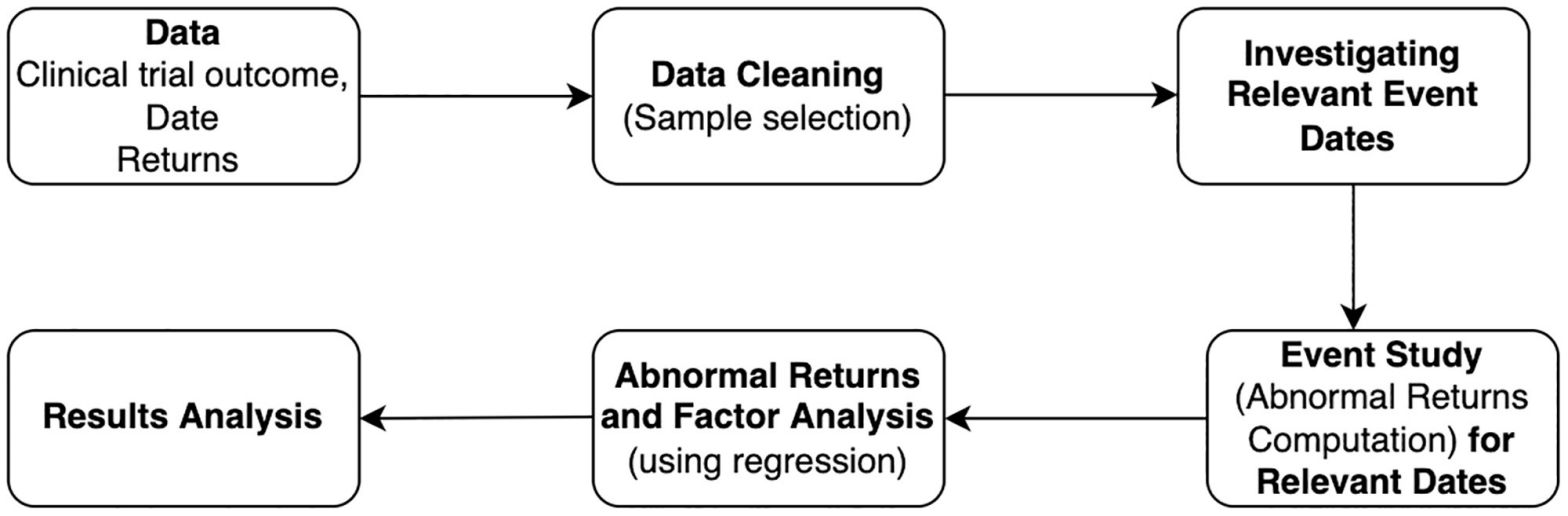
This plot diagrams the steps involved in this event study, from the initial data collection to the analysis of our results.

In the *Data* and *Methodology* sections, we describe the details of the dataset and the methodology of our event study, summarized more concisely in Fig 1. In the *Results and Discussion* section, we present the results and our findings from the study, while the *Robustness Checks* section provides a summary of the tests we perform to validate our findings. We conclude with a discussion about potential extensions.

## Literature review

The event study is a widely used method of analysis to quantify the financial effects of an event on the price movements of a stock. One of the earliest published event studies was a 1933 student performed for measuring the impact of a stock split [8]. A study by *Fama et al*. [9] proposed a methodology that is in essence the same as that used today, although over the years, it has been modified and refined for different use cases. For example, [10] deals with the issues in performing an event study for daily financial data. A number of survey papers [11–13] have summarized the econometrics of the event study and explored the evolution of its methodologies.

A variety of types of events have been analyzed in the literature, including mergers and acquisitions [14–16], earnings announcements [17, 18], news headlines [19], financial reporting [20, 21], and stock messages on the Internet [22]. A detailed review of all the existing event studies in the literature is beyond the scope of this work. Instead, we focus on the event study literature that involves clinical trial results, FDA approval, drug failure, product development, and patent application.

An event study analysis of 24 clinical trial outcomes [23] found that there were asymmetric market reactions to positive and negative outcomes, i.e., underperformance due to negative events was greater in magnitude than the reaction to positive events. Unlike our findings, they conclude that the magnitude of abnormal returns did not differ by phase or firm classification. Another event study [6] consisting of 98 phase 3 trials and 49 products undergoing FDA advisory panel review sought evidence of insider trading, and found indirect evidence that it was common within the biotechnology sector. A study on the long-term returns around clinical trial results (that is, within 120 days of the event) focused on oncology drugs [24], while another [25] analyzed the market overreaction to clinical trial outcome. Focusing on biotechnology product development for 43 phase-3 trial outcomes, [26] analyzed the relationship between abnormal returns and company size, R&D expenditure, and type of result, while [27] analyzed the impact of drug features (e.g., disease and application type) and company features (e.g., size) on the value of abnormal returns.

Event study analyses have also been conducted on FDA approvals [28], pharmaceutical product development [29], drug failures [30], earnings surprises and product news [31], patent infringement [32], and patent application and publication dates in the pharmaceutical industry [33]. More recently, the influence of news of COVID-19 research and development on the biopharmaceutical industries has been analyzed [34], as has the impact of COVID-19 on the stock market [35] and the Indian pharmaceutical industry [36]. S.2 Fig in S1 File summarizes these literatures.

Our work focuses specifically on clinical trial outcomes. While some earlier work focuses on clinical trial results, their scope is limited to due to their very small sample sizes (typically fewer than 150 events), while the events span a much shorter time period. Neither does this earlier work analyze the relationship of clinical trial features like phase, trial design, or accrual on abnormal returns. With over 13,000 clinical trial outcomes spanning a 21-year period, the scope of our event study analysis is orders of magnitude larger than those of previous studies, which translates into more accurate inferences about the impact of clinical trial outcomes on stock’s price movements. Moreover, we also analyze how the impact of clinical trial outcomes on sponsor company stock price movements differs over time.

## Data

We use Citeline data provided by Informa Pharma Intelligence for details of the clinical trials, their outcomes, and their properties. The dataset combines individual clinical trial information from *Trialtrove* and drug approval data from *Pharmaprojects*. It is one of the world’s most comprehensive dataset of global clinical trials and drug development pipelines. The dataset consists of trials sponsored by government, academia, cooperative groups, and industry. We remove trials that are not sponsored by industry. Next, we filter out clinical trials based on multiple criteria: missing event dates, missing reasons for termination, non-availability of returns data for sponsor companies, and ambiguity about the sponsors. Ambiguity in sponsorship refers to unclear information about the sponsor company in the dataset. Furthermore, we filter trials based on valid outcomes or early termination reasons, including only those trials that belong to one of the following categories: safety/adverse effect, lack of efficacy, early positive outcome, positive outcome/primary endpoints met, and negative outcome/primary endpoints not met. To analyze the different outcome types more intuitively, we group trials from different categories as follows: a negative finding includes primary endpoints not met and lack of efficacy; a positive finding includes an early positive outcome and primary endpoints met; closed early includes a lack of efficacy and safety/adverse effect; and full maturity includes primary endpoints met, early positive outcomes and primary endpoints not met.

After all our filters, the final sample sizes of trials are presented in S.1 Table in S1 File. We also include the trial distribution across different phases (1, 2, 3, 4, 1/2, 2/3, 3/4), sponsor company types (biotechnology vs pharmaceutical classification), therapeutic categories (9 categories), and trial design (28 in total). The filtering process resulted in a sample of 13,807 trials, and this process is described in more detail in S.1 Fig in S1 File.

We obtain the sponsor company classification from [37], who classify companies using a *k*-nearest-neighbors clustering algorithm into the following categories on the basis of research and size of the company. Big Pharmaceutical (BP) company research traditionally includes (but is not limited to) small molecules, and BP companies have numerous products that reach patients. Small Pharmaceutical (SP) companies have fewer assets than big pharmaceutical companies, and tend to produce drugs for niche diseases. These companies also have products on the market. Early Biotechnology (EB) companies differ from pharma companies in that their production process typically uses living material, but they may also produce small molecules. EB companies may or may not have drugs in the market. Late Biotechnology (LB) companies are larger in size than EB companies, with a product in market. In the following work, we use the categories EB, LB, SP and BP to classify the companies.

In addition to Citeline dataset, we obtained daily stock returns of the trial sponsor companies from the Center for Research in Security Prices (CRSP). We merged the Citeline and CRSP dataset using fuzzy string matching and manual verification of the correct match on sponsor and company names.

## Methodology

An event study formally consists of multiple components: event definition, stock selection, model definition, model estimation, empirical results, and interpretation. In our analysis, we first define the day of the ‘event’ (the trial outcome declaration date) from the relevant clinical trial dates. Following this step, the stocks of the sponsor companies are selected for the study. For this analysis, we use the Fama–French five-factor model to estimate abnormal returns (The analysis using other factor models is available in the S1 File) Finally, after calculating the abnormal returns, we attribute them to different properties of the clinical trials and sponsor companies.

### Event dates

For a given clinical trial, there are multiple important dates related to trial outcomes. They include:

- **Primary Completion Date**: The date when the final subject was examined or received intervention for data collection for evaluating the primary endpoints of a clinical trial.
- **Primary Endpoint Reported Date**: The earliest date of public report of results that address the primary endpoints of the trial. Reports of interim or preliminary results do not constitute a primary endpoint reported date.
- **Publish Date**: The earliest date of result publication, which includes interim and preliminary results.
- **Enrollment Close Date**: The date on which the study completed patient enrollment.

The most movement in the company returns should happen on the day when information about trial outcomes is released to the public for the first time. Later in our results, we will show how different dates are relevant for different trial outcomes.

### Sample selection

The companies sponsoring the trials are most directly affected by the outcomes of the clinical trials; hence, we select these sponsor companies for our analysis. In some cases, there is an ambiguity in sponsorship, which happens when sponsor companies merge with, or are acquired by, other companies during the course of trial. In such cases, we identify the correct sponsor company to use for the event study by looking at the merger or acquisition date.

### Abnormal returns model

In most event studys, it is conventionally assumed that asset returns are jointly multivariate normal in distribution, and independently and identically distributed over time. These assumptions allow us to develop various simple models for the estimation of abnormal returns prior to, on, or after the day of the event. We use eventstudy python package for the estimation procedures, which are described in more detail in [38].

The Fama–French five-factor model [39] determines the relationship between different factor returns and security returns:

$$\begin{array}{c} R_{i,t}=\alpha_{i}+\beta_{i,1}*R_{m,t}+\beta_{i,2}*R_{SMB,t}+\beta_{i,3}*R_{HML,t}+\beta_{i,4}*R_{RMW,t}+\beta_{i,5}*R_{CMA,t}+\epsilon_{i,t},\\ E\left[\epsilon_{i,t}\right]=0,Var\left[\epsilon_{i,t}\right]=\sigma_{\epsilon_{i}}^{2} \end{array}$$

where *R*_*i*,*t*_ represents the returns of asset *i* at time *t*; *R*_*m*,*t*_, *R*_*SMB*,*t*_, *R*_*HML*,*t*_, *R*_*RMW*,*t*_, and *R*_*CMA*,*t*_ are the factor returns at time *t*; and *α*_*i*_, *β*_*i*,1_, *β*_*i*,2_, *β*_*i*,3_, *β*_*i*,4_, *β*_*i*,5_, and $\sigma_{\epsilon_{i}}^{2}$ are the factor loadings or parameters.

**Fig 2:**
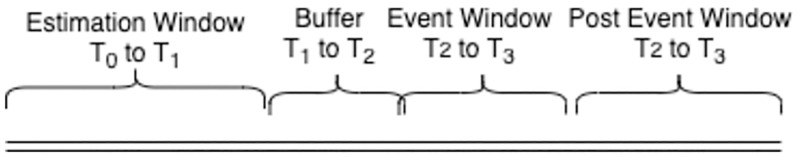
Timeline of the event. *T*_0_ to *T*_1_ is the window used for model estimation. A buffer window is kept between the estimation and the event window to prevent any information leakage. The event window is *T*_2_ to *T*_3_, so that the day of the event *t* = 0 lies within the event window.

The timeline of an event is shown in Fig 2. The model parameters can be estimated using the observed returns of the security and factors during the estimation period. The estimation window in our study was 252 trading days (i.e., one year). Once the model’s parameters are estimated, they are used to obtain the returns in the event window, $\hat{R_{i,t}}$, and abnormal returns (AR) are calculated as the difference between the observed and estimated returns, ($R_{i,t}-\hat{R_{i,t}}$). When multiple (*N*) events are analyzed together, the average abnormal returns (AAR) (we use the terms ‘abnormal returns’ and ‘average abnormal returns’ interchangeably in the remaining analysis.) are given by:

$$\begin{array}{c} AAR=\frac{\Sigma AR}{N}. \end{array}$$

### Return attribution

To measure the impact of clinical trial and sponsor company properties on the stock prices of the sponsor companies, we perform a regression of abnormal returns on these properties:

$$\begin{array}{c} Y_{i}=\alpha +\beta *phase+\gamma *company_{type}+\delta *outcome+\eta *disease+\theta *targetAcc.+\zeta *design, \end{array}$$ (1)

where the regressors are coded using one-hot encoding, except for *targetAcc*., which is a continuous variable. The sign of abnormal returns of unsuccessful outcomes (e.g., a primary endpoint not met, an adverse effect, or a lack of efficacy) are inverted to make them comparable with positive outcomes. The regressors are:

$$\begin{array}{cc} phase: & \text{clinical}\;\text{trial}\;\text{phase}\\ company_{type}: & \text{sponsor}\;\text{company}\;\text{type}\\ outcome: & \text{clinical}\;\text{trial}\;\text{outcome}\\ disease: & \text{therapeutic}\;\text{care}\;\text{classification}\;\text{associated}\;\text{with}\;\text{a}\;\text{trial}\\ targetAcc.: & \text{target}\;\text{accrual}\;\text{for}\;\text{a}\;\text{trial}\\ design: & \text{clinical}\;\text{trial}\;\text{design} \end{array}$$

*Y*_*i*_ represents the abnormal returns, and *i* indicates the various time windows around the event date for which the abnormal returns were calculated: *i* = −1 for the day prior to the event window; *i* = 0 for the day of the event; *i* = 0 to 1 for the day of and day after the event; and *i* = 2 for two days after the event.

Since *Y*_*i*_ are abnormal returns, which are estimated with some error, we use bootstrapping to compute the confidence intervals associated with the coefficients of the regressions.

## Results and discussion

In this section, we first present the identification of the most significant date for various clinical outcomes, then analyze the abnormal returns for a two-week period before and after the events, and discuss the relationship between abnormal returns and clinical trial properties. Finally, we include examples of the average abnormal returns due to clinical trial outcomes conditional on multiple factors.

### Clinical trial outcome dates

Depending on the outcome of the trial, different dates may be significant for clinical trials. We first determine the dates associated with clinical trials that have the largest impact on the stock price of the companies. To do so, we segregate trials on the basis of reason for termination and analyze them separately. We run a multi-event study for the trials with the following event dates: the primary completion date, the date when the primary endpoints were reported, the earliest date of result publication, and the enrollment close date. We present the abnormal returns on the day of the event, day 0 and the cumulative abnormal returns on day 0 and 1, along with the standard errors in Table 1.

**Table 1:** Average abnormal returns for different outcome types on various significant dates.

Outcome	Day	Primary Completion	Endpoint	Publish	Close
Safety/Adverse Effect	0	−**0.75** (0.09)	0.08 (0.11)	−0.39 (0.09)	−0.40 (0.10)
	0,1	−**0.82** (0.12)	0.25 (0.15)	−0.72 (0.13)	−0.26 (0.14)
Lack of Efficacy	0	−**1.70** (0.09)	−0.28 (0.09)	−0.45 (0.07)	−1.58 (0.10)
	0,1	−**2.43** (0.13)	−0.32 (0.13)	−0.47 (0.11)	−2.37 (0.15)
Early Positive Outcome	0	**5.31** (0.20)	0.03 (0.24)	0.38 (0.25)	0.05 (0.21)
	0,1	**6.35** (0.28)	0.48 (0.33)	1.07 (0.35)	0.41 (0.30)
Primary Endpoints Met	0	−0.02 (0.02)	0.15 (0.02)	**0.46** (0.02)	0.04 (0.02)
	0,1	−0.01 (0.03)	0.12 (0.03)	**0.54** (0.03)	0.08 (0.03)
Primary Endpoints Not Met	0	−0.20 (0.04)	−0.33 (0.05)	−**1.97** (0.04)	0.06 (0.04)
	0,1	−0.19 (0.06)	−0.49 (0.07)	−**2.71** (0.06)	0.17 (0.06)

Average abnormal returns on day of the event (day 0) and cumulative average abnormal returns (day 0,1) for trials with different outcomes on significant dates with their standard error (bracket). Day 0 abnormal returns are in the first row and day 0,1 abnormal returns are in the second row for each outcome. We find that for trial outcomes of safety/adverse effect, lack of efficacy, and early positive outcome, the highest abnormal returns are obtained on the primary completion date. For completed trials with outcomes of primary endpoint met or not met, the highest magnitude of abnormal returns are obtained on the earliest date of result publication. The maximum absolute abnormal returns are in bold.

We observe that the magnitude of average abnormal returns for an early positive outcome are greater on the primary completion date than for any other date. This implies that, as soon as the data from the final subject has been collected and the results appear promising, companies will release the results, so that the largest abnormal returns are observed on the primary completion date. However, when the outcome is that the primary endpoint is met or not met, we find that the value of average abnormal returns is greatest on the earliest date of result publication (the publish date). On this date, companies release their interim results after a thorough study of the data collected thus far.

Trials that show a safety/adverse effect or lack of efficacy have the largest magnitude of abnormal returns on their primary completion date, but these are comparable to the abnormal returns obtained on the enrollment close date and the publish date. Trials that show a safety/adverse effect or lack of efficacy outcome are terminated early, and development is stopped. In the case of a safety/adverse effect, for a majority of trials, the primary completion date is earlier than the publish date. Since details about the termination of the trial are available on the primary completion date, we therefore observe the largest abnormal returns on the primary completion date. For trials that show a lack of efficacy, we observe the same primary completion and enrollment close date for many trials. This explains the higher abnormal returns on the enrollment close date.

For the remainder of this study, we use the primary completion date as the event date for the following outcomes: a safety/adverse effect, a lack of efficacy, and an early positive outcome. These outcomes constitute 9% of the dataset. For the remaining results, when primary endpoints are met or not met, we use the publish date as the event date. These outcomes constitute 91% of the dataset.

### Abnormal returns

**Fig 3:**
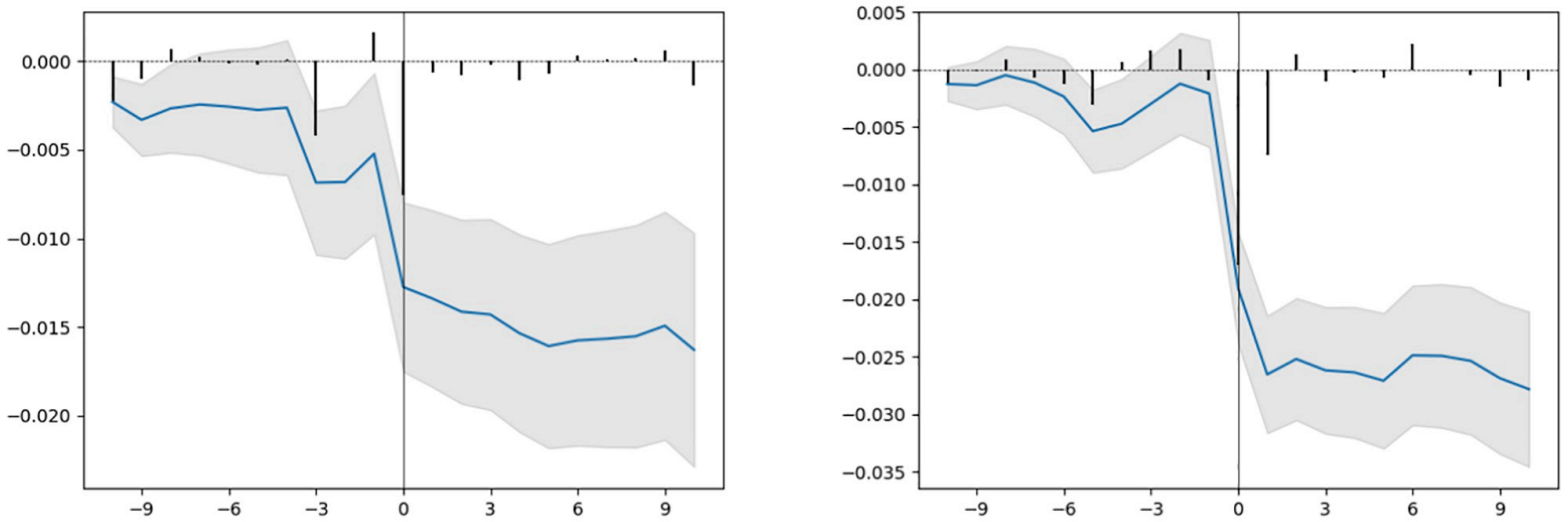
Left: Safety and adverse effect, Right: Lack of efficacy.

**Fig 4:**
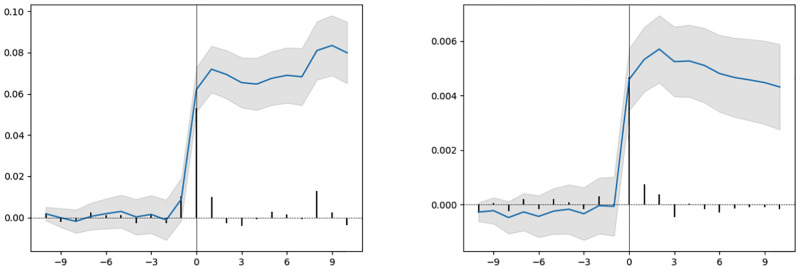
Left: Early positive outcome, Right: Primary endpoints met.

**Fig 5:**
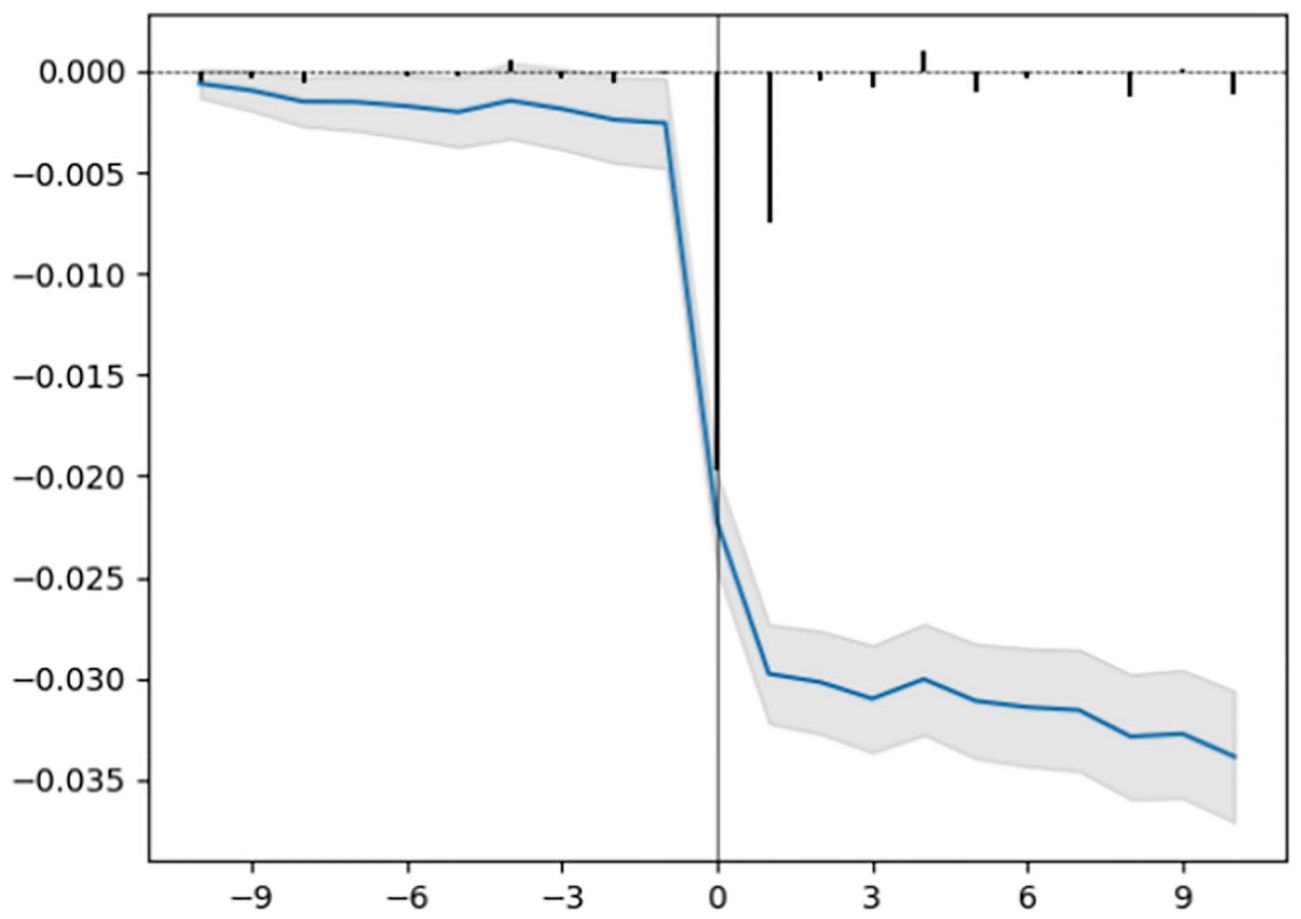
Primary endpoints not met.

In Figs 3–5, we include the average abnormal returns and the cumulative average abnormal returns for trial outcomes for the two-week trading period before and after the event date. As expected, we observe high average abnormal returns on the day of the event (day 0 and day 1). For trials that declare their results after trading hours, the movement in stock prices is expected to happen on the day after the result declaration date. For unsuccessful trial outcomes such as a safety/adverse effect, a lack of efficacy, or primary endpoints not met, we observe cumulative abnormal returns decreasing during the two-week period after the event date.

In Figs 3–5, we observe variation in average abnormal returns. Even within each outcome type, we find a huge spread in the abnormal returns of individual events. For a safety/adverse effect, lack of efficacy and primary endpoints not met outcomes, the minimum value of abnormal returns for individual events is −80%, −90%, and −100% respectively. However, for the early positive outcome and primary endpoints met, the maximum value of abnormal return is 137% and 485%, respectively, which is quite different from the average abnormal returns. Such variation in abnormal returns for individual events within each outcome category may potentially be due to the properties of the clinical trials and sponsor companies involved in the trials, including the trial phase, company classification, type of result, therapeutic area of the disease, and trial design.

### Abnormal returns and trial properties

We conjecture that the impact of a trial outcome on the sponsor’s stock prices is dependent on specific properties of a clinical trial and its sponsor company. From S.1 Table in S1 File, we can observe that the distributions of trials across different phases and sponsor company types are different. Hence, to measure the impact of these trial and company properties on the stock prices of the sponsor companies, we perform a regression of abnormal returns on these different properties, as given by Eq (1). For trials with negative outcomes, we reverse the sign of the abnormal returns prior to regression.

Average abnormal returns and cumulative average abnormal returns with a 95% confidence interval for a multi-event study of trials with different outcomes. The x-axis represents the day (0 is the day of the event) and the y-axis represents the abnormal return values (scale: fraction).

**Fig 6:**
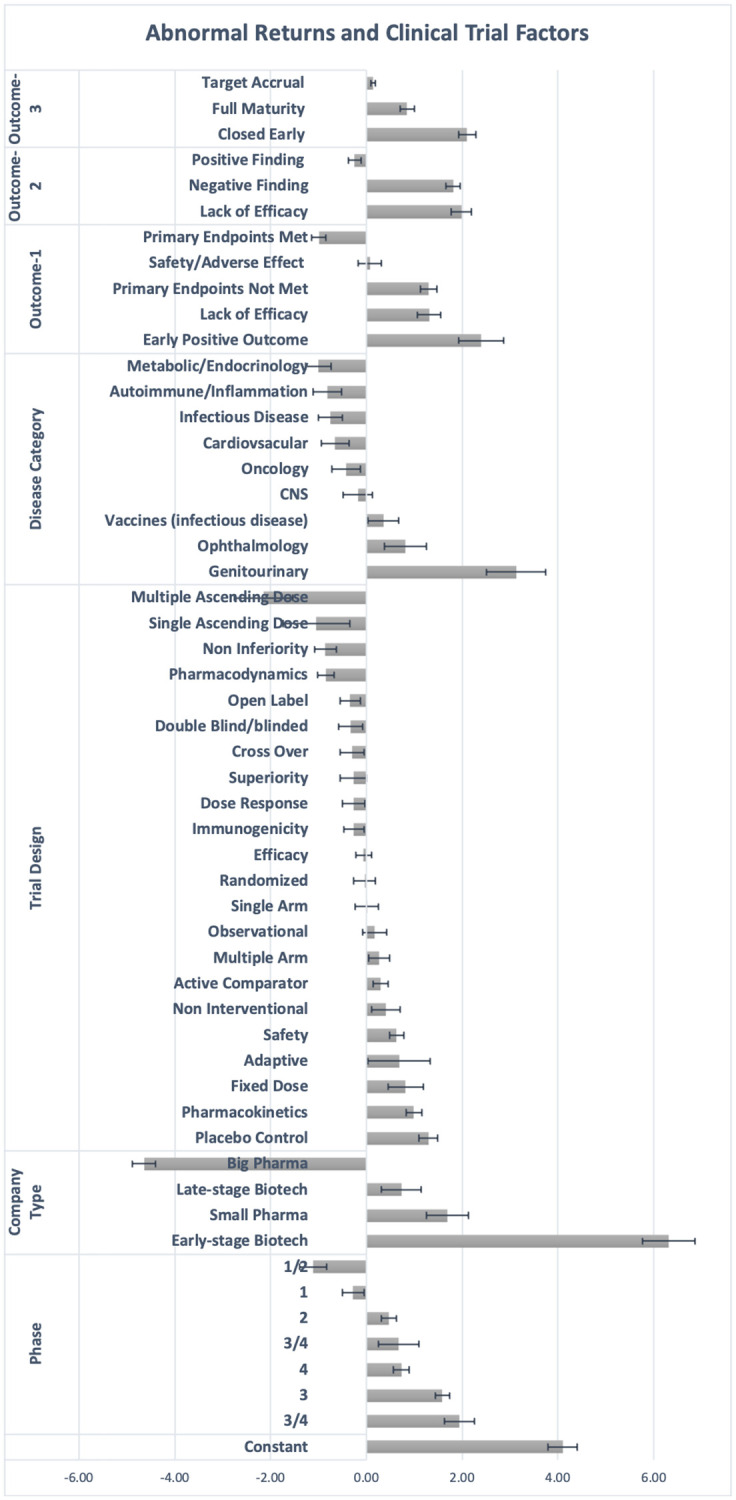
This bar plot presents the relative abnormal returns due to different clinical trial and sponsor company factors on the day of event (day 0–1). These abnormal returns are obtained from the regression coefficients using Eq (1) on the day of event (day 0 and day 1). The x-axis is in percentage points and the 95% confidence intervals are plotted on the bar plots.

In Tables 2–8, we present the abnormal returns for various properties (given by the regression coefficients) with their standard errors on days around the event, and analyze them in the following section. In Fig 6, we present the contribution of clinical trial properties on the average abnormal return of a company on the day of the event (day 0–1) in a bar plot. We then discuss each trial or company property and its abnormal return individually.

**Table 2:** Outcome types.

Outcome	*δ*_−1_(SE)	*δ*_0_ (SE)	*δ*_0−1_ (SE)	*δ*_2_ (SE)
Early Positive Outcome	0.18 (0.17)	2.21 (0.17)	2.40 (0.24)	0.08 (0.17)
Lack of Efficacy	0.09 (0.09)	0.57 (0.09)	1.31 (0.12)	-0.23 (0.09)
Primary Endpoints Not Met	-0.01 (0.06)	0.79 (0.06)	1.30 (0.09)	0.1 (0.06)
Safety/Adverse Effect	-0.22 (0.09)	-0.01 (0.09)	0.07 (0.13)	0.11 (0.09)
Primary Endpoints Met	0.0 (0.05)	-0.74 (0.05)	-0.99 (0.08)	0.0 (0.05)
Lack of Efficacy	0.09 (0.08)	1.17 (0.08)	1.99 (0.11)	-0.17 (0.08)
Negative Finding	-0.05 (0.05)	1.27 (0.05)	1.81 (0.07)	0.15 (0.05)
Positive Finding	0.00 (0.05)	-0.08 (0.05)	-0.24 (0.06)	0.05 (0.05)
Closed Early	0.00 (0.06)	1.33 (0.07)	2.11 (0.09)	-0.05 (0.06)
Full Maturity	0.02 (0.05)	0.64 (0.05)	0.85 (0.08)	0.08 (0.05)

**Table 3:** Target accrual.

	*θ*_−1_(SE)	*θ*_0_ (SE)	*θ*_0−1_ (SE)	*θ*_2_ (SE)
Target Accrual	0.016 (0.015)	0.158 (0.015)	0.144 (0.022)	0.021 (0.015)

**Table 4:** Company sponsor category.

Company Type	*γ*_−1_(SE)	*γ*_0_ (SE)	*γ*_0−1_ (SE)	*γ*_2_
Early-stage Biotech	−0.33 (0.20)	4.65 (0.2)	6.31 (0.28)	0.36 (0.20)
Small Pharma	0.12 (0.15)	1.63 (0.15)	1.69 (0.22)	0.12 (0.15)
Late-stage Biotech	0.32 (0.15)	0.31 (0.15)	0.73 (0.21)	−0.21 (0.15)
Big Pharma	−0.02 (0.09)	−3.67 (0.09)	−4.64 (0.12)	−0.08 (0.09)

**Table 5:** Phase categories.

Phase	*β*_−1_(SE)	*β*_0_ (SE)	*β*_0−1_ (SE)	*β*_2_ (SE)
2/3	−0.15 (0.11)	1.63 (0.11)	1.95 (0.16)	0.09 (0.11)
3	0.03 (0.06)	0.99 (0.05)	1.59 (0.08)	−0.04 (0.06)
4	−0.01 (0.06)	0.39 (0.06)	0.73 (0.08)	0.04 (0.06)
3/4	−0.13 (0.15)	0.32 (0.16)	0.68 (0.22)	−0.10 (0.16)
2	0.05 (0.06)	0.30 (0.06)	0.47 (0.08)	0.14 (0.06)
1	0.15 (0.08)	−0.33 (0.08)	−0.28 (0.12)	0.06 (0.08)
1/2	−0.07 (0.10)	−0.64 (0.10)	−1.11 (0.14)	0.07 (0.10)

**Table 6:** Disease therapeutic category.

Disease	*η*_−1_(SE)	*η*_0_ (SE)	*η*_0−1_ (SE)	*η*_2_ (SE)
Genitourinary	0.09 (0.22)	1.51 (0.22)	3.13 (0.32)	0.25 (0.22)
Ophthalmology	0.10 (0.16)	0.33 (0.16)	0.81 (0.23)	0.11 (0.16)
Vaccines (Infectious Disease)	0.04 (0.12)	−0.09 (0.12)	0.35 (0.16)	0.09 (0.12)
CNS	0.07 (0.11)	0.25 (0.11)	−0.18 (0.16)	0.04 (0.11)
Oncology	−0.02 (0.11)	−0.14 (0.11)	−0.43 (0.15)	−0.10 (0.11)
Cardiovascular	0.09 (0.10)	−0.01 (0.10)	−0.65 (0.15)	0.04 (0.10)
Infectious Disease	−0.02 (0.09)	−0.49 (0.09)	−0.76 (0.13)	−0.13 (0.09)
Autoimmune/Inflammation	0.07 (0.11)	−0.39 (0.11)	−0.81 (0.15)	−0.04 (0.11)
Metabolic/Endocrinology	0.02 (0.10)	−0.51 (0.10)	−1.00 (0.14)	0.03 (0.10)

**Table 7:** Clinical trial design.

Design	*ζ*_−1_(SE)	*ζ*_0_ (SE)	*ζ*_0−1_ (SE)	*ζ*_2_ (SE)
Placebo Control	-0.09 (0.07)	1.10 (0.07)	1.30 (0.10)	0.14 (0.07)
Pharmacokinetics	-0.03 (0.06)	0.46 (0.06)	0.99 (0.08)	-0.07 (0.06)
Fixed Dose	0.01 (0.14)	0.68 (0.13)	0.82 (0.19)	0.10 (0.13)
Adaptive	0.45 (0.24)	0.35 (0.23)	0.69 (0.33)	-0.32 (0.23)
Safety	-0.03 (0.06)	0.57 (0.06)	0.63 (0.08)	0.20 (0.05)
Non Interventional	0.02 (0.11)	0.44 (0.11)	0.40 (0.15)	-0.32 (0.11)
Active Comparator	0.06 (0.05)	0.05 (0.05)	0.30 (0.08)	0.08 (0.05)
Multiple Arm	0.05 (0.08)	0.25 (0.08)	0.26 (0.11)	-0.03 (0.08)
Observational	0.01 (0.09)	0.22 (0.09)	0.17 (0.13)	0.02 (0.09)
Single Arm	-0.03 (0.09)	0.14 (0.09)	0.00 (0.12)	-0.03 (0.09)
Randomized	-0.1 (0.08)	0.14 (0.08)	-0.04 (0.12)	-0.08 (0.08)
Efficacy	-0.04 (0.06)	-0.18 (0.06)	-0.06 (0.09)	-0.09 (0.06)
Immunogenicity	0.01 (0.08)	0.21 (0.08)	-0.26 (0.11)	0.02 (0.08)
Dose Response	-0.01 (0.09)	-0.07 (0.09)	-0.26 (0.12)	-0.03 (0.08)
Superiority	0.02 (0.10)	-0.11 (0.10)	-0.27 (0.14)	0.08 (0.1)
Cross Over	-0.01 (0.09)	-0.15 (0.09)	-0.30 (0.13)	-0.04 (0.09)
Double blind/blinded	0.06 (0.09)	-0.04 (0.09)	-0.33 (0.13)	-0.17 (0.09)
Open Label	0.03 (0.08)	-0.02 (0.08)	-0.34 (0.11)	-0.03 (0.08)
Pharmacodynamics	-0.01 (0.06)	-0.63 (0.06)	-0.85 (0.09)	0.03 (0.06)
Non Inferiority	-0.39 (0.08)	-0.64 (0.08)	-0.86 (0.12)	-0.01 (0.08)
Single Ascending Dose	0.16 (0.25)	-0.43 (0.25)	-1.05 (0.36)	0.41 (0.25)
Multiple Ascending Dose	-0.09 (0.23)	-1.94 (0.22)	-2.14 (0.31)	-0.17 (0.22)

**Table 8:** Regression constant.

	*α*_−1_(SE)	*α*_0_ (SE)	*α*_0−1_ (SE)	*α*_2_ (SE)
Constant	0.04 (0.11)	2.83 (0.11)	4.10 (0.16)	0.06 (0.11)

#### Clinical trial phase

Clinical trials are conducted in phases, with each phase designed to answer specific questions. Phase 1 is designed to evaluate the safety of the treatment, phase 2 is designed to evaluate the effectiveness of treatment in a small and carefully curated population, and phase 3 trials are generally designed to compare the effectiveness and safety in a larger and more heterogenous population against the existing standard treatment. Typically, therapeutics obtain FDA approval after a successful phase 3 trial, although they may go to a phase 4 trial following approval, in which the long-term side effects of the new therapeutic are studied. In many cases, different trial phases are combined to speed up the development and testing process.

Statistics for the abnormal returns for each phase category (keeping other factors constant) are given in Table 2. We observe that phase 2/3 trials have the greatest impact on the stock returns of sponsor companies, followed by phase 3 trials, while phase 1 and 1/2 trials have the least impact on abnormal returns. On the day of the event, day 0 and day 0–1, phase 2/3 trials have higher abnormal returns than phase 1/2 trials by 2.27% and 3.06%, respectively.

Phase 3 trials are generally costly and require significant capital [40], and the results of phase 3 trials determine whether a therapeutic can be commercialized and used by patients. This explains the higher abnormal returns for phase 3 and 2/3 trials. Even though phase 4 trials happen after FDA approval, negative findings from such trials raise questions about the long-term effectiveness of the therapeutic; hence, phase 4 trials had higher abnormal returns as well.

#### Company type

A large number of biotechnology and pharmaceutical companies are involved in the development of new therapeutics as clinical trial sponsors. The success or failure of these clinical trials has an impact on the business of the sponsor company, leading to changes in its stock prices.

We present the statistics of abnormal returns by sponsor type in S.12 Table in S1 File. As observed, the abnormal returns for early biotechnology companies are the largest, followed by small pharmaceutical companies, with large pharmaceutical companies having the smallest abnormal returns. Early biotech companies had 8.32% and 10.95% higher abnormal returns than large pharmaceutical companies on the day of the event—day 0 and day 0–1—respectively.

Early-stage biotechnology companies tend to be smaller in size, and are involved in earlier stage discovery and development efforts for specific potential therapeutics. In general, they do not have FDA-approved products. The trial results for a sponsored therapeutic candidate thus have a large impact on the growth of the company. Hence, we observe higher values of abnormal returns for these companies. At the same time, large pharmaceutical companies have a higher market capitalization and tend to have multiple products on the market; hence, the results of clinical trial outcomes have the least impact on this category of companies. Similarly, late-stage biotechnology companies are established companies with FDA-approved products already on the market, and thus clinical trial results have less of an impact on their stock market returns.

Moreover, we observe that the sponsor company classification has the biggest impact on abnormal returns compared to other properties of clinical trials.

#### Outcome type

As mentioned in earlier sections, the trials analyzed in our sample have very different outcomes. Table 1 shows the average abnormal returns for these different outcomes on various dates. As observed, the abnormal returns for unsuccessful outcomes are negative, while those for successful trials are positive. In the regression in Eq (1), we invert the sign of unsuccessful outcomes to compare them directly to successful outcomes. Here, we analyze the magnitude of abnormal returns controlling for trial properties.

In Table 2, we present the abnormal returns by outcome type. We find that trial results with early positive outcomes have the largest abnormal returns, followed by unsuccessful outcomes like unmet primary endpoints and a lack of efficacy. The outcome of primary endpoints met have the lowest value of abnormal returns. The difference between abnormal returns for trials with an early positive outcome and trials with successful endpoints are 2.95% and 3.39%, respectively, on day 0 and day 0–1. The high abnormal returns for early positive outcomes may be due to the surprise factor associated with an earlier than anticipated declaration of results. Higher abnormal returns for trials with unsuccessful outcomes than for trials with successful outcomes (asymmetric market reaction) is consistent with earlier findings in the literature [23].

To analyze the different outcome types more intuitively, we create new groups from the dataset’s original categories as follows: a negative finding includes primary endpoints not met and a lack of efficacy; a positive finding includes an early positive outcome and primary endpoints met; a finding of closed early combines a lack of efficacy and safety/adverse effect; and full maturity combines primary endpoints met, early positive outcomes and primary endpoints not met. We find that the magnitude of abnormal returns of clinical trials due to a negative finding is higher than a positive finding, consistent with earlier results in the literature [23]). Similarly, trials that are closed early have a higher magnitude of abnormal returns than trials reaching full maturity.

#### Target accrual

Target accrual is the number of subjects that are planned to participate in the study. A larger target accrual signifies a larger investment by the company for the clinical trial, and a treatment tested on a larger number of subjects has more reliable results. We include the coefficient for target accrual in Table 3. We observe that trials with larger targeted accruals have a higher value of abnormal returns. More specifically, an increase in the targeted accrual of a trial by 1,000 subjects, keeping the other properties constant, produces a higher value of abnormal returns by 0.158% on the day of the event.

#### Disease

Clinical trials test potential treatments for diseases belonging to many different therapeutic areas. In our data, these include: genitourinary, ophthalmology, vaccines (for infectious disease), CNS, oncology, cardiovascular, infectious disease, autoimmune/inflammation, and metabolic/endocrinology. By including the disease type as one of the regressors, we are able to analyze the impact of disease category on a company’s abnormal stock returns.

In Table 7, we include the average abnormal returns by disease category. We find that genitourinary diseases have the largest abnormal returns, (the higher abnormal returns are due to multiple trial outcomes following the investigation of BioSante Pharmaceuticals), followed by ophthalmology and vaccine trials. The abnormal returns are smallest for endocrinology and autoimmune disease trials. It is widely observed that oncology drugs are best-sellers; however, contrary to this stylized fact, we do not observe a high level of abnormal returns for oncology trial outcomes, despite oncology trials being the largest disease category in our dataset. Nevertheless, the abnormal returns for oncology trials are higher than those for infectious disease, autoimmune, and endocrinology trials.

#### Design

There are multiple ways of designing clinical trials, controlling for cost, to establish the effect of a potential new therapeutic [41]. This trial design depends on the characteristics of treatment, clinical endpoints, trial phase, the availability of a control group, funding, and other properties.

In Table 7, we present the abnormal returns for different trial designs (in our regression, we exclude design categories that have fewer than 100 trials). We find that placebo-controlled trials have the largest abnormal returns, followed by pharmacokinetic, fixed dose, adaptive, and safety trials. Meanwhile, designs like single and multiple ascending dose, pharmacodynamic, and non-inferiority trial designs have the smallest abnormal returns. A placebo-controlled, double-blind trial design is considered the gold standard in determining the effect of a treatment and used for designing phase 3 trials. This potentially explains the higher abnormal returns of placebo-controlled trials.

We also observe that competing trial designs have varying abnormal returns. For example, for an open-label trial design, where the subject and researchers know that the subject is receiving a treatment, compared to a double-blinded trial design, where neither the subject nor the researchers know who is receiving a treatment or a placebo, the abnormal returns are not statistically different. Meanwhile, the single ascending dose trial design has higher abnormal returns than the multiple ascending dose design. Similarly, the superiority trial design (showing that a new method is better than an active control or placebo) has higher abnormal returns than non-inferiority trial designs (showing that a new method is not inferior to an active control or placebo).

### Stock movement given trial properties

As discussed earlier, the linear factor model for abnormal stock returns in Eq 1 can be used by investment managers to obtain average abnormal returns for the stock of a sponsor company at the declaration dates of a clinical trial result. This is demonstrated by the following examples.

The regression coefficients from Tables 2–8 can be used to compare the abnormal returns of clinical trials with different properties. For example, phase 2/3 trials sponsored by early-stage biotech (EB) companies with an early positive outcome have 13.54% higher absolute abnormal returns than phase 1/2 trials with a big pharmaceutical (BP) company sponsor and an outcome of primary endpoints met on the day of the event. Similarly, the cumulative abnormal returns for two days (day 0–1) for a clinical trial with an early positive outcome (2.56%) and a target accrual of one thousand subjects (0.134%), sponsored by an early biotechnology company (6.33%), at phase 3 of the trial (1.60%) for a treatment for genitourinary disease (2.85%) using a double-blinded, placebo-controlled trial design (1.23%–0.33%) are 18.49%. This information has several potentially useful applications for investment managers and other stakeholders.

The unexplained average abnormal returns after the regression are presented in Table 8. As expected, the average of the unexplained abnormal returns are not statistically significant prior to the day of the event (day −1) or after the event (day 2). The value of *α* increases from 2.83% on the event date (day 0) to 4.10% for the day after the event (including the event date). The higher value of unexplained abnormal returns (*α*) implies there are factors apart from the ones included in this study that might explain the abnormal returns of these companies.

We find that abnormal returns on the day before the event (*δ*_−1_, *θ*_−1_, *γ*_−1_, *β*_−1_, *η*_−1_, *ζ*_−1_, *α*_−1_) are not significant. Similarly, the abnormal returns are not significant on the day after the event (*δ*_2_, *θ*_2_, *γ*_2_, *β*_2_, *η*_2_, *ζ*_2_, *α*_2_), implying that markets incorporate the information related to clinical trials, and the sponsor stock prices stabilize.

We also analyze the pattern of abnormal returns of the clinical trial outcomes over time. The discussion and results are included in the S1 File (in the section *Abnormal Returns Over Time* and S.2 Table in S1 File).

In Tables 2 to 8, we present the regression coefficients obtained using Eq (1) for the abnormal returns (computed using the French-Fama 5-factor model) for the day prior to the event date (−1), on the day of the event (0 and 0–1), and the day after the event date (2). Rows in each table are the different trial properties, and the columns are the regressed coefficients for different days around the event (−1, 0, 0 − 1, 2). These coefficients can be interpreted as the average abnormal returns observed for the sponsor company for a trial outcome with that specific property, controlling for other properties of the trial. The abnormal returns are statistically insignificant prior to and after the event date (day −1 and day 2), while they are significant for properties on the days of the event (day 0 and day 0–1).

## Robustness checks

We perform several robustness checks to determine the sensitivity of our results. In addition to the Fama-French 5-factor model for computing the abnormal returns in the event study, we also apply a constant-mean and market model, which is a single factor model, and the results are in S.3-S.16 Tables in S1 File. We find that the results obtained using the market and constant-mean model are statistically similar to those using the Fama-French 5-factor model presented in Tables 2–8.

We also estimate a return attribution model using a subset of our dataset that includes only two outcome categories: when primary endpoints were met (a positive outcome), and when primary endpoints were not met (a negative outcome). These categories use the earliest date of result publication as their event date, and the results are presented in S.17-S.23 Tables in S1 File. Our findings are consistent with the return attribution model trained on the full dataset.

Standard errors for the abnormal returns due to different factors are computed using 100,000 iterations of the bootstrap process. Each iteration includes sampling the dataset and computing the regression coefficient given by Eq 1. The number of bootstrap iterations is decided by increasing the bootstrap iterations until the average value and standard errors of the regression coefficients converge.

The observations discussed in *Results and Discussion* are based on the statistically significant results of our analysis. As we vary the parameters of the event study such as the length of the estimation and buffer windows (as in Fig 2), the results remain statistically similar.

## Conclusion

In this study, we analyze the impact of clinical trial outcomes on the stock prices of their sponsor companies using one of the largest clinical trials datasets so far constructed, with 13,807 clinical trial outcomes (after filtering) from 2000 to 2020. Given the large size of the dataset and its two-decade span, the findings should represent reasonably accurate measures of the stock-price impact of clinical trial outcomes for sponsor companies.

We identify dates specific to the individual clinical trial’s timeline on which the largest movements in the stock’s abnormal returns are observed. By fixing the date of that event, we are able to attribute the abnormal returns to clinical trial properties such as outcome type, target accrual, phase, disease therapeutic category, trial design, and sponsor company classification. The attribution of returns to trial properties is performed for abnormal returns on the day of the event and the days after/prior to the event (i.e., days −1, 0, 1, and 2). In the attribution process, we uncover multiple findings about the abnormal returns for various trial properties.

As observed from the significant *α* in our regression, we find that the clinical trial properties we use cannot completely explain the average abnormal returns observed due to clinical trial outcomes. This implies there are additional factors influencing the company’s stock price movements apart from the documented trial properties. One such property may be the “importance” or “buzz” surrounding a clinical trial. For example, the importance of Moderna’s mRNA vaccine trial for protection against SARS-CoV-2 was much higher than a clinical trial for any other disease at the same time. Such importance could be captured by analyzing the news and sentiment indexes associated with the trial. In future research, we plan to extend this analysis to quantify the hype or sentiment associated with clinical trials and correlate it with their abnormal returns.

## Supporting information

S1 File (PDF)
