# Supplementary material for: The reaction of sponsor stock prices to clinical trial outcomes: An event study analysis
Source: PLoS One. 2022 Sep 2;17(9):e0272851. doi: 10.1371/journal.pone.0272851 (PMC9439234; doi:10.1371/journal.pone.0272851)
Supplement: S1 File — (PDF) [file pone.0272851.s001.pdf]

Supporting information

S1. Data

The Citeline dataset contains a large number of records about clinical trials performed in the recent past. However, it has significant missing data issues. To use this data for analysis, we perform multiple data cleaning steps, summarized in Figure S.1. Our first step removes all trials that have a missing reason for termination or reasons for termination with an ambiguity in event dates. The termination reasons with ambiguity in their event date include poor enrollment, lack of funding, and business decisions, such as a drug strategy shift or pipeline reprioritization.

From the remaining 46,003 trials, we remove those trials with missing event dates. Since an event study analysis can only be performed for public companies with available stock market returns, we remove the trials that have no stock returns for their sponsors. These sponsor types included academic institutions, government institutions, and private companies.

Next, we exclude those trials from our analysis when confounding factors such as class action lawsuits filed against the company appear in the dataset. Due to the large size of the dataset, it is not possible to isolate all the confounding events. However, some trials are isolated when we are able to find such confounding events. For example, the trials conducted by Repros Therapeutics are not included in our dataset because of the class action lawsuit filed against them on August 8, 2009, that led to abnormal returns around its clinical trial outcome dates.

Finally, we remove those trials with missing clinical trial and sponsor company properties because they are unable to be included in our regression model. After cleaning the dataset, we are left with 13,807 trials for the study. The number of trials across outcomes and trial properties are included in Table S.1.

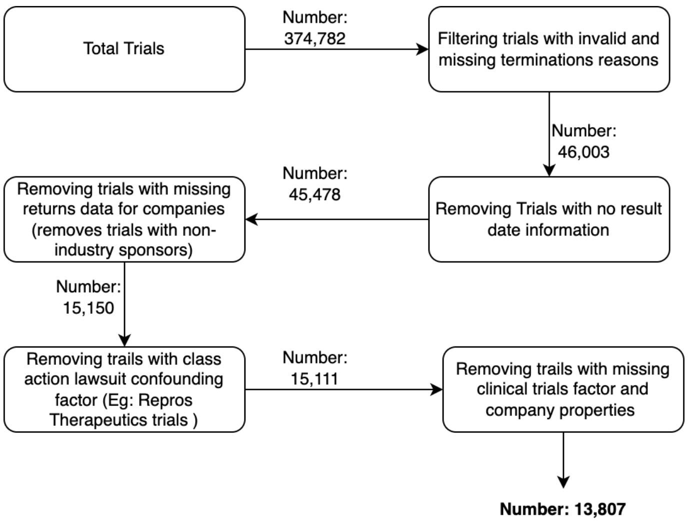

Fig S.1. Data Cleaning Process. The flowchart presents the different steps involved in data cleaning. The number of trials left in the dataset after every step are given above the arrows. The total number of clinical trials used for the study after data cleaning was 13,807.

S2.Results: Abnormal Returns Over Time

Table S.1. Number of Trials

Category	Adverse Effect	Lack of Efficacy	Early +ve Outcome	Primary Endpts. Met	Primary Endpts. not
Total	389	698	127	10156	2437
Phase 1	97	55	12	1060	95
Phase 2	144	364	32	2863	1058
Phase 3	57	168	71	3723	805
Phase 4	18	7	4	1801	297
Phase 1/2	54	77	2	524	99
Phase 2/3	14	26	5	140	63
Phase 3/4	3	0	1	36	18
Big Pharmaceutical	340	568	108	8716	2049
Early-stage Biotechnology	10	28	4	361	105
Late-stage Biotechnology	13	50	11	517	143
Small Pharmaceutical	26	52	4	561	140
Oncology	208	438	73	2626	898
Endocrinology	30	30	11	1815	262
Cardiovascular	33	36	12	869	230
CNS	39	90	14	1431	523
Autoimmune	48	83	10	1815	385
Genitourinary	4	5	0	118	27
Infectious	38	34	6	1454	178
Ophthalmology	3	7	3	177	47
Vaccines	3	6	2	880	58
Randomized	221	420	99	6564	1996
Efficacy	284	612	110	8539	2197
Safety	339	599	114	8903	2021
Placebo Control	132	258	58	3514	1221
Pharmacokinetics	183	308	59	2861	652
Pharmacodynamics	104	189	23	1648	380
Adaptive	10	29	1	96	33
Fixed dose	8	8	0	197	67
Bioequivalence	0	1	0	63	2
Bioavailability	5	0	2	40	2
Cross over	27	25	18	659	164
Drug-drug interaction	6	3	3	53	7
Double-blind/blinded	154	289	73	4760	1433
Open label	239	430	60	5483	1026
Superiority	0	20	15	383	107
Active comparator	53	142	39	2455	667
Non-Inferiority	1	11	1	669	68
Dose Response	46	74	11	878	199
Single Ascending Dose	19	10	2	121	7
Multiple Ascending Dose	12	13	1	145	13
Immunogenicity	23	61	10	1254	157
Observational	4	6	3	564	52
Non-Interventional	1	1	0	227	11
Single Arm	139	234	22	2660	376
Multiple Arm	202	417	88	6167	1800
Basket	1	0	0	10	2
Umbrella	0	0	0	5	5
Patient Registry	1	1	0	34	3

In this table, we present the number of trials across different outcomes and properties. The rows labeled phase 1, 2, 3, 4, 1/2, 2/3, and 3/4 represent the different phases of the clinical trial process, while rows containing biotechnology and pharmaceutical represent the types of the sponsor company. The next nine rows are for the disease therapeutic category of the clinical trial, and the remaining rows are for the clinical trial design. The total number of trials in the dataset is 13,807.

In this section, we perform the regression given by Eq. (1), adding an extra variable for the year on the right-hand side. The year variable is one-hot encoded,

taking values from 2001 to 2020. The event dates for the clinical trial were used to obtain the year.

Table S.2 presents the average abnormal returns for different years as determined by the regression. We observed a pattern of increasing abnormal returns during the period of the financial crisis of 2007–2009, and during 2014 to 2017. We found that trials in 2007 had the largest abnormal returns, while trials in 2006 had the lowest abnormal returns.

Years	Day 0 (SE)	Day 0-1 (SE)
2001	−0.54 (0.46)	−0.17 (0.64)
2002	0.22 (0.36)	−0.95 (0.51)
2003	−0.73 (0.36)	−0.71 (0.51)
2004	−0.15 (0.23)	−0.38 (0.32)
2005	−0.09 (0.16)	−0.27 (0.23)
2006	−0.56 (0.15)	−1.11 (0.21)
2007	1.64 (0.11)	2.10 (0.16)
2008	−0.10 (0.08)	−0.15 (0.12)
2009	0.21 (0.11)	0.38 (0.16)
2010	−0.90 (0.08)	−0.95 (0.12)
2011	−0.10 (0.07)	0.30 (0.10)
2012	0.00 (0.08)	0.07 (0.11)
2013	−0.12 (0.08)	0.19 (0.11)
2014	0.40 (0.08)	0.61 (0.12)
2015	0.82 (0.08)	1.43 (0.12)
2016	0.99 (0.09)	1.78 (0.13)
2017	0.36 (0.09)	0.54 (0.13)
2018	0.29 (0.10)	−0.06 (0.14)
2019	0.02 (0.11)	−0.13 (0.15)
2020	0.02 (0.13)	−0.17 (0.18)

Table S.2. In this table, we present the regression coefficients for each year included in our dataset. In 2007, 2015, 2016, and 2017, the abnormal returns for clinical trials were higher, while 2006 and 2010 had small abnormal returns for clinical trial outcomes.

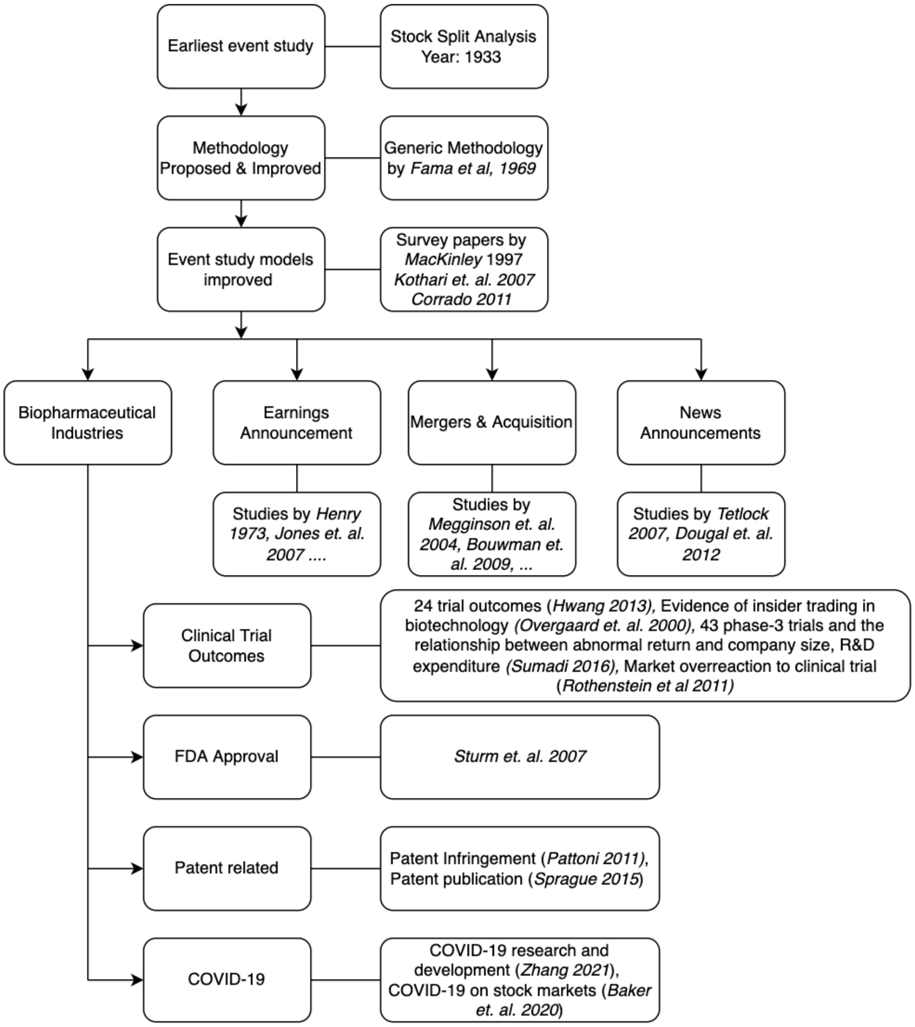

Fig S.2. Literature Review Summary.

Table S.3. Outcome types

Outcome	δ_{-1} (SE)	δ_0 (SE)	δ_{0-1} (SE)	δ_2 (SE)
Early Positive Outcome	0.29 (0.21)	1.99 (0.21)	2.31 (0.30)	-0.01 (0.21)
Lack of Efficacy	0.06 (0.11)	0.68 (0.11)	1.38 (0.15)	-0.23 (0.11)
Primary Endpoints Not Met	0.02 (0.08)	0.87 (0.08)	1.33 (0.11)	0.12 (0.08)
Safety/Adverse Effect	-0.31 (0.11)	0.00 (0.11)	0.06 (0.15)	0.14 (0.11)
Primary Endpoints Met	-0.01 (0.07)	-0.76 (0.07)	-1.08 (0.09)	-0.02 (0.06)
Lack of Efficacy	0.06 (0.09)	1.22 (0.09)	2.03 (0.13)	-0.19 (0.09)
Negative Finding	-0.02 (0.06)	1.29 (0.06)	1.81 (0.09)	0.15 (0.06)
Positive Finding	0.00 (0.05)	-0.16 (0.05)	-0.36 (0.08)	0.01 (0.05)
Closed Early	-0.04 (0.08)	1.35 (0.08)	2.12 (0.11)	-0.07 (0.08)
Full Maturity	0.04 (0.06)	0.59 (0.06)	0.76 (0.09)	0.04 (0.06)

Table S.4. Target Accrual

	θ_{-1} (SE)	θ_0 (SE)	θ_{0-1} (SE)	θ_2 (SE)
Target Accrual	0.023 (0.021)	0.169 (0.021)	0.149 (0.030)	0.026 (0.021)

Table S.5. Company sponsor category

Company Type	γ_{-1} (SE)	γ_0 (SE)	γ_{0-1} (SE)	γ_2
Early-stage Biotech	-0.31 (0.22)	4.61 (0.23)	6.26 (0.32)	0.38 (0.23)
Small Pharma	0.15 (0.17)	1.62 (0.17)	1.79 (0.25)	0.02 (0.17)
Late-stage Biotech	0.30 (0.17)	0.32 (0.17)	0.60 (0.24)	-0.16 (0.17)
Big Pharma	-0.02 (0.10)	-3.66 (0.10)	-4.62 (0.14)	-0.12 (0.10)

Table S.6. Phase categories.

Phase	β_{-1} (SE)	β_0 (SE)	β_{0-1} (SE)	β_2 (SE)
2/3	-0.06 (0.14)	1.47 (0.14)	1.74 (0.20)	0.12 (0.14)
3	0.09 (0.07)	1.08 (0.07)	1.65 (0.09)	-0.03 (0.07)
4	0.01 (0.07)	0.48 (0.07)	0.87 (0.10)	0.08 (0.07)
3/4	-0.14 (0.19)	0.36 (0.19)	0.60 (0.28)	-0.22 (0.19)
2	0.12 (0.07)	0.31 (0.07)	0.48 (0.09)	0.12 (0.07)
1	0.2 (0.09)	-0.38 (0.09)	-0.32 (0.13)	0.10 (0.09)
1/2	-0.04 (0.11)	-0.64 (0.11)	-1.03 (0.16)	0.01 (0.11)

Table S.7. Disease therapeutic category

Disease	η_{-1} (SE)	η_0 (SE)	η_{0-1} (SE)	η_2 (SE)
Genitourinary	0.07 (0.27)	1.60 (0.28)	3.44 (0.39)	0.17 (0.28)
Ophthalmology	0.12 (0.19)	0.32 (0.19)	0.75 (0.27)	0.25 (0.19)
Vaccines (Infectious Disease)	-0.04 (0.13)	-0.07 (0.13)	0.33 (0.19)	0.17 (0.13)
CNS	0.10 (0.13)	0.23 (0.13)	-0.12 (0.18)	0.15 (0.13)
Oncology	-0.06 (0.12)	-0.18 (0.12)	-0.40 (0.17)	0.01 (0.12)
Cardiovascular	0.13 (0.12)	0.07 (0.12)	-0.52 (0.17)	0.04 (0.12)
Infectious Disease	0.07 (0.11)	-0.47 (0.11)	-0.61 (0.15)	-0.09 (0.11)
Autoimmune/Inflammation	0.04 (0.12)	-0.31 (0.12)	-0.71 (0.17)	0.01 (0.12)
Metabolic/Endocrinology	-0.03 (0.11)	-0.52 (0.11)	-0.96 (0.16)	0.08 (0.11)

Table S.8. Clinical Trial Design

Design	ζ_{-1} (SE)	ζ_0 (SE)	ζ_{0-1} (SE)	ζ_2 (SE)
Placebo Control	-0.03 (0.08)	1.12 (0.08)	1.38 (0.11)	0.11 (0.08)
Pharmacokinetics	-0.02 (0.07)	0.50 (0.07)	1.0 (0.1)	-0.07 (0.07)
Adaptive	0.53 (0.26)	0.48 (0.26)	0.80 (0.37)	-0.34 (0.26)
Safety	-0.09 (0.07)	0.60 (0.07)	0.71 (0.09)	0.25 (0.07)
Fixed Dose	-0.03 (0.16)	0.52 (0.16)	0.68 (0.23)	0.12 (0.16)
Active Comparator	0.14 (0.07)	0.09 (0.07)	0.41 (0.09)	0.11 (0.07)
Non Iinterventional	-0.05 (0.14)	0.48 (0.13)	0.32 (0.19)	-0.27 (0.13)
Multiple Arm	0.02 (0.09)	0.20 (0.09)	0.17 (0.13)	-0.08 (0.09)
Observational	0.02 (0.11)	0.11 (0.11)	0.07 (0.16)	0.09 (0.11)
Randomized	-0.11 (0.09)	0.14 (0.09)	0.02 (0.14)	-0.05 (0.09)
Single Arm	-0.08 (0.10)	0.14 (0.10)	-0.02 (0.14)	-0.11 (0.1)
Efficacy	-0.06 (0.07)	-0.19 (0.07)	-0.08 (0.10)	-0.05 (0.07)
Immunogenicity	-0.03 (0.09)	0.24 (0.09)	-0.16 (0.12)	0.04 (0.09)
Dose Response	-0.01 (0.1)	-0.05 (0.1)	-0.21 (0.14)	-0.05 (0.1)
Cross Over	-0.11 (0.11)	-0.08 (0.11)	-0.27 (0.15)	-0.10 (0.11)
Superiority	-0.02 (0.11)	-0.14 (0.11)	-0.28 (0.16)	0.08 (0.11)
Open Label	0.07 (0.09)	-0.06 (0.09)	-0.42 (0.13)	-0.05 (0.09)
Double blind/blinded	0.03 (0.11)	-0.10 (0.11)	-0.46 (0.15)	-0.19 (0.11)
Pharmacodynamics	0.01 (0.07)	-0.60 (0.08)	-0.77 (0.11)	0.10 (0.07)
Non Inferiority	-0.35 (0.1)	-0.68 (0.10)	-0.93 (0.14)	-0.01 (0.1)
Single Ascending Dose	0.13 (0.28)	-0.41 (0.28)	-0.96 (0.39)	0.28 (0.28)
Multiple Ascending Dose	-0.12 (0.25)	-1.86 (0.25)	-1.90 (0.36)	-0.13 (0.25)

Table S.9. Regression Constant

	α_{-1} (SE)	α_0 (SE)	α_{0-1} (SE)	α_2 (SE)
Constant	0.04 (0.13)	2.78 (0.13)	4.01 (0.18)	-0.01 (0.13)

In Tables S.3 to S.9, we present the regression coefficients obtained using Eq. (1) for the abnormal returns (computed using **constant mean** model) for the day prior to the event date (-1), on the day of the event (0 and 0–1), and the day after the event date (2). The rows are the trial properties and columns are the coefficients for the days around the event(-1, 0, 0-1 , 2). The coefficients can be interpreted as the average abnormal returns observed for the sponsor company for a trial outcome with a specific property, controlling for other properties of the trial. The abnormal returns are statistically insignificant prior to and after the event date (day -1 and day 2), while they are significant for properties on the days of the event (day 0 and day 0-1).

Table S.10. Outcome types

Outcome	δ_{-1} (SE)	δ_0 (SE)	δ_{0-1} (SE)	δ_2 (SE)
Early Positive Outcome	0.14 (0.17)	1.59 (0.17)	1.83 (0.24)	-0.02 (0.17)
Primary Endpoints Not Met	-0.03 (0.06)	0.9 (0.07)	1.35 (0.09)	0.11 (0.06)
Lack of Efficacy	0.08 (0.09)	0.62 (0.09)	1.34 (0.13)	-0.22 (0.09)
Safety/Adverse Effect	-0.17 (0.09)	0.21 (0.09)	0.22 (0.13)	0.12 (0.09)
Primary Endpoints Met	-0.01 (0.05)	-0.57 (0.06)	-0.81 (0.08)	0.03 (0.05)
Lack of Efficacy	0.08 (0.08)	1.11 (0.08)	1.91 (0.11)	-0.19 (0.08)
Negative Finding	-0.05 (0.05)	1.29 (0.05)	1.76 (0.08)	0.14 (0.05)
Positive Finding	-0.01 (0.05)	-0.04 (0.05)	-0.19 (0.07)	0.06 (0.05)
Closed Early	0.0 (0.07)	1.32 (0.07)	2.05 (0.09)	-0.07 (0.07)
Full Maturity	0.0 (0.06)	0.66 (0.06)	0.86 (0.08)	0.08 (0.06)

Table S.11. Target Accrual

	θ_{-1} (SE)	θ_0 (SE)	θ_{0-1} (SE)	θ_2 (SE)
Target Accrual	0.017 (0.016)	0.171 (0.016)	0.154 (0.023)	0.015 (0.016)

Table S.12. Company sponsor category

Company Type	γ_{-1} (SE)	γ_0 (SE)	γ_{0-1} (SE)	γ_2
Early-stage Biotech	-0.37 (0.22)	4.52 (0.22)	6.42 (0.31)	0.29 (0.22)
Small Pharma	0.13 (0.16)	1.71 (0.16)	1.77 (0.23)	0.13 (0.16)
Late-stage Biotech	0.32 (0.16)	0.33 (0.16)	0.54 (0.22)	-0.17 (0.16)
Big Pharma	-0.01 (0.09)	-3.65 (0.09)	-4.68 (0.13)	-0.11 (0.09)

Table S.13. Phase categories.

Phase	β_{-1} (SE)	β_0 (SE)	β_{0-1} (SE)	β_2 (SE)
3/4	-0.10 (0.12)	1.45 (0.12)	1.83 (0.17)	0.13 (0.12)
3	0.05 (0.06)	1.03 (0.06)	1.60 (0.08)	-0.02 (0.06)
3/4	-0.15 (0.16)	0.35 (0.16)	0.74 (0.23)	-0.08 (0.16)
4	-0.04 (0.06)	0.39 (0.06)	0.69 (0.08)	0.06 (0.06)
2	0.04 (0.06)	0.27 (0.06)	0.39 (0.08)	0.15 (0.06)
1	0.16 (0.08)	-0.33 (0.08)	-0.38 (0.12)	0.04 (0.08)
1/2	-0.11 (0.1)	-0.55 (0.10)	-1.00 (0.15)	-0.04 (0.11)

Table S.14. Disease therapeutic category

Disease	η_{-1} (SE)	η_0 (SE)	η_{0-1} (SE)	η_2 (SE)
Genitourinary	0.11 (0.23)	1.63 (0.23)	3.62 (0.32)	0.20 (0.23)
Ophthalmology	0.16 (0.16)	-0.10 (0.16)	0.40 (0.23)	0.21 (0.16)
Vaccines (Infectious Disease)	0.02 (0.12)	-0.19 (0.12)	0.35 (0.17)	0.11 (0.12)
CNS	0.1 (0.11)	0.27 (0.11)	0.16 (0.16)	0.06 (0.11)
Oncology	0.03 (0.11)	-0.22 (0.11)	-0.26 (0.15)	-0.06 (0.11)
Cardiovascular	0.14 (0.11)	-0.05 (0.11)	-0.53 (0.15)	0.02 (0.11)
Autoimmune/Inflammation	0.12 (0.11)	-0.42 (0.11)	-0.59 (0.16)	-0.04 (0.11)
Infectious Disease	0.07 (0.09)	-0.54 (0.09)	-0.66 (0.13)	-0.13 (0.09)
Metabolic/endocrinology	0.06 (0.1)	-0.58 (0.1)	-0.87 (0.14)	0.09 (0.1)

Table S.15. Clinical Trial Design

Design	ζ_{-1} (SE)	ζ_0 (SE)	ζ_{0-1} (SE)	ζ_2 (SE)
Placebo Control	-0.09 (0.07)	1.11 (0.07)	1.23 (0.1)	0.15 (0.07)
Adaptive	0.46 (0.24)	0.63 (0.24)	1.0 (0.34)	-0.43 (0.24)
Pharmacokinetics	-0.04 (0.06)	0.45 (0.06)	1.0 (0.09)	-0.09 (0.06)
Fixed Dose	-0.01 (0.14)	0.6 (0.14)	0.7 (0.2)	0.04 (0.14)
Safety	-0.05 (0.06)	0.6 (0.06)	0.69 (0.08)	0.21 (0.06)
Active Comparator	0.07 (0.06)	0.14 (0.06)	0.48 (0.08)	0.09 (0.06)
Non Iinterventional	0.01 (0.11)	0.44 (0.11)	0.38 (0.16)	-0.29 (0.11)
Multiple Arm	0.03 (0.08)	0.17 (0.08)	0.18 (0.11)	-0.04 (0.08)
Observational	0.01 (0.09)	0.13 (0.09)	0.05 (0.13)	0.0 (0.09)
Randomized	-0.1 (0.08)	0.13 (0.08)	-0.06 (0.12)	-0.09 (0.08)
Efficacy	-0.03 (0.06)	-0.19 (0.06)	-0.1 (0.09)	-0.08 (0.06)
Single Arm	-0.06 (0.09)	0.06 (0.09)	-0.1 (0.13)	-0.03 (0.09)
Immunogenicity	-0.01 (0.08)	0.29 (0.08)	-0.16 (0.11)	0.06 (0.08)
Cross Over	-0.06 (0.09)	-0.09 (0.09)	-0.3 (0.13)	-0.06 (0.09)
Superiority	-0.02 (0.1)	-0.21 (0.1)	-0.36 (0.14)	0.09 (0.1)
Double blind/blinded	0.06 (0.09)	-0.07 (0.09)	-0.36 (0.13)	-0.17 (0.09)
Dose Response	0.05 (0.09)	-0.13 (0.09)	-0.36 (0.12)	-0.02 (0.09)
Open Label	0.05 (0.08)	0.0 (0.08)	-0.4 (0.11)	-0.03 (0.08)
Pharmacodynamics	0.0 (0.07)	-0.61 (0.07)	-0.77 (0.09)	0.09 (0.07)
Non Inferiority	-0.35 (0.09)	-0.7 (0.09)	-0.97 (0.12)	-0.04 (0.08)
Single Ascending Dose	0.13 (0.26)	-0.57 (0.26)	-1.13 (0.37)	0.41 (0.26)
Multiple Ascending Dose	-0.16 (0.23)	-1.71 (0.23)	-1.66 (0.33)	-0.14 (0.23)

Table S.16. Regression Constant

	α_{-1} (SE)	α_0 (SE)	α_{0-1} (SE)	α_2 (SE)
Constant	0.02 (0.12)	2.75 (0.12)	3.93 (0.17)	0.03 (0.12)

In Tables S.10 to S.16, we present the regression coefficients obtained using Eq. (1) for the abnormal returns (computed using **market** model) for the day prior to the event date (-1), on the day of the event (0 and 0–1), and the day after the event date (2). Rows are the properties in each table, and columns are the coefficients for the days around the event(-1, 0, 0-1 , 2). The coefficients can be interpreted as the average abnormal returns observed for the sponsor company for a trial outcome with a specific property, controlling for other properties of the trial. The abnormal returns are statistically insignificant prior to and after the event date (day -1 and day 2) while they are significant for properties on the days of the event (day 0 and day 0-1).

Table S.17. Outcome types

Outcome	δ_{-1} (SE)	δ_0 (SE)	δ_{0-1} (SE)	δ_2 (SE)
Primary Endpoints not Met (Negative Outcome)	0.01 (0.06)	1.78 (0.06)	2.64 (0.08)	0.08 (0.06)
Primary Endpoints Met (Positive Outcome)	0.02 (0.05)	0.24 (0.05)	0.31 (0.07)	-0.02 (0.05)

Table S.18. Target Accrual

	θ_{-1} (SE)	θ_0 (SE)	θ_{0-1} (SE)	θ_2 (SE)
Target Accrual	0.020 (0.016)	0.226 (0.016)	0.217 (0.023)	0.009 (0.017)

Table S.19. Company sponsor category

Company Type	γ_{-1} (SE)	γ_0 (SE)	γ_{0-1} (SE)	γ_2
Early-stage Biotech	-0.29 (0.21)	4.24 (0.21)	6.03 (0.29)	0.33 (0.21)
Small Pharma	0.12 (0.17)	1.75 (0.16)	1.40 (0.23)	0.24 (0.16)
Late-stage Biotech	0.27 (0.16)	-0.43 (0.16)	-0.17 (0.22)	-0.21 (0.16)
Big Pharma	0.00 (0.09)	-3.46 (0.09)	-4.33 (0.13)	-0.15 (0.09)

Table S.20. Phase categories.

Phase	β_{-1} (SE)	β_0 (SE)	β_{0-1} (SE)	β_2 (SE)
2/3	-0.12 (0.13)	1.80 (0.13)	1.99 (0.18)	0.00 (0.13)
3	0.01 (0.06)	0.67 (0.06)	1.12 (0.08)	0.00 (0.06)
2	0.06 (0.06)	0.23 (0.06)	0.41 (0.08)	0.11 (0.06)
4	-0.02 (0.06)	0.10 (0.06)	0.29 (0.08)	0.04 (0.06)
3/4	-0.09 (0.16)	0.02 (0.16)	0.22 (0.22)	-0.03 (0.15)
1	0.16 (0.09)	-0.32 (0.09)	-0.25 (0.13)	0.05 (0.09)
1/2	-0.1 (0.12)	-0.79 (0.12)	-1.17 (0.16)	0.07 (0.12)

Table S.21. Disease therapeutic category

Disease	η_{-1} (SE)	η_0 (SE)	η_{0-1} (SE)	η_2 (SE)
Genitourinary	0.05 (0.23)	1.67 (0.23)	3.49 (0.32)	0.36 (0.23)
Ophthalmology	0.08 (0.16)	0.21 (0.16)	0.79 (0.23)	0.19 (0.16)
Vaccines (Infectious Disease)	0.06 (0.12)	-0.13 (0.12)	0.19 (0.17)	0.18 (0.12)
CNS	0.00 (0.12)	0.24 (0.12)	-0.14 (0.17)	0.16 (0.12)
Infectious Disease	-0.07 (0.09)	-0.67 (0.10)	-0.85 (0.14)	-0.04 (0.10)
Oncology	-0.05 (0.11)	-0.52 (0.11)	-0.89 (0.16)	0.08 (0.11)
Autoimmune/Inflammation	0.04 (0.11)	-0.56 (0.11)	-0.94 (0.16)	0.06 (0.11)
Cardiovascular	0.00 (0.11)	-0.33 (0.11)	-0.94 (0.15)	0.14 (0.11)
Metabolic/Endocrinology	0.01 (0.10)	-0.72 (0.10)	-1.18 (0.14)	0.17 (0.10)

Table S.22. Clinical Trial Design

Design	ζ_{-1} (SE)	ζ_0 (SE)	ζ_{0-1} (SE)	ζ_2 (SE)
Placebo Control	-0.08 (0.07)	1.11 (0.07)	1.37 (0.10)	0.04 (0.08)
Pharmacokinetics	-0.04 (0.06)	0.59 (0.06)	0.92 (0.09)	-0.03 (0.06)
Safety	-0.05 (0.06)	0.48 (0.06)	0.70 (0.08)	0.04 (0.06)
Fixed Dose	0.00 (0.13)	0.39 (0.13)	0.57 (0.18)	0.12 (0.13)
Active Comparator	0.06 (0.06)	0.19 (0.06)	0.37 (0.08)	0.14 (0.06)
Non Iinterventional	0.02 (0.11)	0.39 (0.11)	0.33 (0.15)	-0.30 (0.11)
Observational	0.02 (0.09)	0.25 (0.09)	0.18 (0.13)	0.04 (0.09)
Multiple Arm	0.06 (0.08)	0.17 (0.08)	0.16 (0.12)	0.04 (0.08)
Open Label	0.07 (0.08)	0.22 (0.08)	-0.07 (0.12)	-0.04 (0.08)
Efficacy	-0.05 (0.06)	-0.12 (0.06)	-0.07 (0.09)	-0.05 (0.06)
Randomized	-0.09 (0.09)	0.06 (0.09)	-0.11 (0.12)	-0.14 (0.09)
Dose Response	0.01 (0.09)	0.02 (0.09)	-0.13 (0.13)	-0.06 (0.09)
Single Arm	-0.04 (0.09)	0.06 (0.09)	-0.13 (0.13)	0.00 (0.09)
Superiority	-0.03 (0.10)	-0.03 (0.10)	-0.14 (0.14)	0.02 (0.10)
Adaptive	0.41 (0.27)	0.06 (0.26)	-0.16 (0.38)	-0.54 (0.27)
Double blind/blinded	0.08 (0.10)	0.13 (0.09)	-0.26 (0.13)	-0.05 (0.10)
Immunogenicity	-0.01 (0.08)	0.10 (0.08)	-0.35 (0.11)	0.00 (0.08)
Cross Over	0.01 (0.10)	-0.40 (0.10)	-0.57 (0.14)	-0.03 (0.10)
Single Ascending Dose	0.01 (0.29)	-0.07 (0.28)	-0.60 (0.40)	0.52 (0.29)
Non Inferiority	-0.37 (0.08)	-0.60 (0.08)	-0.73 (0.12)	-0.05 (0.08)
Pharmacodynamics	0.0 (0.07)	-0.73 (0.07)	-0.83 (0.1)	0.00 (0.07)
Multiple Ascending Dose	-0.08 (0.24)	-1.99 (0.24)	-2.31 (0.34)	-0.13 (0.24)

Table S.23. Regression Constant

	α_{-1} (SE)	α_0 (SE)	α_{0-1} (SE)	α_2 (SE)
Constant	0.03 (0.10)	2.01 (0.10)	2.95 (0.14)	0.06 (0.10)

In Tables S.17 to S.23, we present the regression coefficients obtained using Eq. (1) for the abnormal returns (computed using the Fama-French 5-factor model) for the day prior to the event date (-1), on the day of the event (0 and 0–1), and the day after the event date (2). The abnormal return attribution model is built using a subset of dataset, including only two outcomes: primary endpoints met (positive outcome) and primary endpoints not met (negative outcome). Rows are the properties in each table and columns are the coefficients for the days around the event (-1, 0, $0 - 1$, 2). The coefficients can be interpreted as the average abnormal returns observed for the sponsor company for a trial outcome with that specific property, controlling for other properties of the trial. The abnormal returns are statistically insignificant prior to and after the event date (day -1 and day 2), while they are significant for properties on the days of the event (day 0 and day 0-1).
